# Positive Impact of Organized Physical Exercise on Quality of Life and Fatigue in Children and Adolescents With Cancer

**DOI:** 10.3389/fped.2021.627876

**Published:** 2021-06-07

**Authors:** Filippo Spreafico, Francesco Barretta, Michele Murelli, Marco Chisari, Giovanna Gattuso, Monica Terenziani, Andrea Ferrari, Laura Veneroni, Cristina Meazza, Maura Massimino

**Affiliations:** ^1^Department of Medical Oncology and Hematology, Pediatric Oncology Unit, Fondazione IRCCS Istituto Nazionale dei Tumori, Milan, Italy; ^2^Medical Statistics, Biometry and Bioinformatics Unit, Fondazione IRCCS Istituto Nazionale dei Tumori, Milan, Italy; ^3^Palliative Care, Pain Therapy and Rehabilitation Unit, Fondazione IRCCS Istituto Nazionale dei Tumori, Milan, Italy

**Keywords:** patient-reported outcomes, health-related quality of life, exercise, physical activity, pediatric cancer, cancer-related fatigue

## Abstract

**Background:** Cancer and its treatment can cause serious health issues that impair physical and mental well-being in children and adolescents. Exercise may be a valid strategy for managing some symptoms, including fatigue. In the light of our experience, we provide further justification for including exercise as part of routine childhood cancer care.

**Methods:** Forty-four children and adolescents who had solid cancers not contraindicating their movement were invited to join an in-hospital 6-week supervised exercise program, and asked afterwards to complete validated quality of life and fatigue scales. The program consisted of personalized workout sessions of aerobic, resistance and flexibility exercises. The results obtained on the scales were compared between 21 patients who engaged in the exercise program (GYM group) and 23 who refused (No-GYM group), examining the different dimensions of health-related quality of life (physical, emotional, cognitive, social) and fatigue (general, sleep/rest, cognitive) in the two groups.

**Results:** Being diagnosed with cancer initially prompted all but one of the respondents to drop-out of previous routine exercise or sports although their continuation had not been contraindicated. After 6 weeks of exercise, the GYM group's scores for quality of life and fatigue showed a statistically significant better perceived emotional functioning, and a trend toward a better social functioning than in the No-GYM group.

**Conclusion:** We suggest that exercise improves the satisfaction of children and adolescents with cancer with their physical, mental and social functioning. We would emphasize the potential benefits of general practitioners discussing and recommending exercise for their young patients with cancer.

## Introduction

Children and adolescents with cancer are often treated with chemotherapy, sometimes combined with radiotherapy and/or surgery. We know their disease and its treatments can cause various morbidities, including fatigue, neuropathy, sleep disruption, chronic pain, depression, anxiety, and cognitive impairment (1–3). Overall, the physical functioning of children and adolescents with cancer declines during their treatment, and this can interfere with their quality of life (QoL) (4–6), as well as increasing the risk of certain chronic diseases (2).

There has been growing interest in studying the value of physical exercise in cancer patients, albeit focusing more on adults than on children so far (7–11). Regular high-quality exercise can help patients receiving therapy and cancer survivors to avoid being trapped in a self-perpetuating cycle of deteriorating physical functioning, which can exacerbate the negative consequences of fatigue and a sedentary lifestyle (5, 11–13).

Health-related QoL (HRQoL) is a construct that describes a person's perception of their own physical, mental and social well-being (14–17). Positive associations have been reported between HRQoL and fitness levels in childhood cancer survivors (18, 19). Cancer-related fatigue is a common and distressing condition that significantly lowers the HRQoL of patients with cancer (20, 21), but higher levels of physical activity have been associated with lower levels of cancer-related fatigue (22). The literature on the prevalence of cancer-related fatigue in children and adolescents is contradictory (23). The routine use of systemic pharmacological approaches to combat fatigue in children is generally not recommended (24). The optimal management of fatigue—especially in children and adolescents while on active treatment–remains a clinical challenge (21).

Physical vitality is an important marker of any child's and teenager's physical and psychosocial well-being, be they healthy or ill, and higher levels of physical activity have been found to correlate with better cognitive outcomes (25–28).

The aim of the present study was to assess the effect of an in-hospital training program on the physical vitality and fatigue of children during or after treatment for cancer. The study's findings could help to identify potential barriers to physical activity and participation in sports for children and adolescents with cancer, and to promote exercise programs for children and adolescents with cancer to enhance their physical, psychosocial and emotional functioning.

## Methods

### Participants and Study Design

Between April and August 2018, consecutive patients (aged 5–21 years) diagnosed with solid tumors or lymphomas who were receiving or had completed their treatment, and who had no disabilities and/or morbidities severe enough to prevent any form of physical activity, were personally invited by their oncologists to attend exercise sessions at the hospital gym (29). The sessions were individual and each lasted 1 h. They included combinations of cardiovascular training, strength and endurance exercises, relaxation, and muscle stretching (Supplementary Table 1). The exercises were prescribed by sports professionals to suit patients' capabilities, limitations and preferences. The same professionals also actively supervised the workouts. The sessions were scheduled three times a week for a total of 6 weeks.

Patients involved in the study formed two groups (GYM and No-GYM, based on whether they chose to join the exercise program or not), which were compared in descriptive terms and on HRQoL scores. An *ad hoc* questionnaire was used to obtain information on the patients' physical activity levels and habits prior to being diagnosed with cancer, their personal attitudes to participating in sports, and their knowledge and expectations of the institutional physical exercise program.

Certified questionnaires on QoL and fatigue were administered: the PedsQL-4.0 Generic Core Scales (14, 17) (Italian edition) and PedsQL Multidimensional Fatigue Scale (15, 16, 30) (Italian edition). The 23-item multidimensional PedsQL-4.0 Generic Core Scales (14, 17) comprise 4 scales measuring: (1) physical functioning (eight items); (2) emotional functioning (five items); (3) social functioning (five items); and (4) school functioning (five items). Answers were scored on a five-point rating scale ranging from “never” to “almost always.” The total score was calculated as the average of the scores for each item. Total scores were converted into a score ranging from 0 to 100, with higher scores indicating a better QoL. The 18-item PedsQL Multidimensional Fatigue Scale (15, 16, 30) includes three subscales (with six items each) for general fatigue, sleep/rest fatigue, and cognitive fatigue. Respondents were asked to rate how much of a problem each item had been during the previous month on a five-point Likert scale (0 = never, 1 = almost never, 2 = sometimes, 3 = often, 4 = almost always). The total score was calculated as the average of the scores for each item. Total scores were converted into a score ranging from 0 to 100, with higher scores indicating a lower perception of fatigue.

The questionnaires were administered to the GYM group after participants had completed the 6-week training program, and to the No-GYM group at the baseline.

The results obtained were analyzed using classical descriptive statistics for the study population as a whole, and by group (GYM vs. No-GYM). The scores were calculated with the PedsQL™ 4.0 scoring algorithm, and the medians and interquartile ranges (IQR) were recorded for each item and each dimension, and for total scores (if any), for each PedsQL scale. Higher scores indicated a better QoL.

Differences between the two groups in terms of patients' characteristics, and their scores in the questionnaires (by single dimension and total scores) were assessed with Fisher's exact test or the Wilcoxon test. Possible correlations between total scores (for physical health, psychosocial health, and fatigue) were computed using Pearson's correlation index (Pearson's r).

Consent to participation in the study was obtained from all patients and/or their guardians. The institutional Ethics Committee approved the study and the data protection methods.

## Results

### General Results

Altogether, the study participants included 44 patients: the median age of the sample as a whole was 15.5 years (one 26-year-old was also exceptionally included), and 20/44 patients were female. Twenty-one patients (48%) attended the proposed exercise sessions, and formed the GYM group, while 23 (52%) refused to do so and formed the No-GYM group. The two groups differed statistically as regards type of cancer (*P* 0.034): 6 of 8 patients with brain tumors refused to attend the gym; and all patients with neuroblastoma joined the GYM group (Table 1).

**Table 1:** Patients' characteristics and their answers in the general questionnaire, overall and by group.

	**Overall**		**GYM**		**No-GYM**		**Fisher's exact test**
	***N***	**%**	***N***	**%**	***N***	**%**	***p*-value**
Type of cancer							0.034
Nasopharyngeal carcinoma	1	2.3	1	4.8	0	0.0	
Thyroid carcinoma	1	2.3	0	0.0	1	4.3	
Non-Hodgkin's lymphoma	5	11.4	3	14.3	2	8.7	
Brain tumor	8	18.2	2	9.5	6	26.0	
Neuroblastoma	5	11.4	5	23.8	0	0.0	
Osteosarcoma	8	18.2	5	23.8	3	13.0	
Soft tissue sarcoma	6	13.6	3	14.3	3	13.0	
Ewing sarcoma	6	13.6	2	9.5	4	17.4	
Abdominal desmoplastic tumor	2	4.5	0	0.0	2	8.7	
Wilms tumor	2	4.5	0	0.0	2	8.7	
Patients' age (years)							0.809
5–7	1	2.3	1	4.8	0	0.0	
8–12	12	27.3	6	28.6	6	26.1	
13–17	17	38.6	7	33.3	10	43.5	
18–21	13	29.5	6	28.6	7	30.4	
>21	1	2.3	1	4.8	0	0.0	
Gender							0.771
Male	24	54.5	12	57.1	12	52.2	
Female	20	45.5	9	42.9	11	47.8	
Treatment phase							0.872
During treatment	29	65.9	13	61.9	16	69.6	
During follow-up (off therapy)	15	34.1	8	38.1	7	30.4	
Routine physical exercise before the diagnosis of cancer?							0.548
Yes	27	61.4	14	66.7	13	56.5	
No	17	38.6	7	33.3	10	43.5	
If so, how often?							0.406
Regularly	3	11.1	2	14.3	1	7.7	
Fairly regularly	15	55.6	7	50.0	8	61.5	
Occasionally	7	25.9	5	35.7	2	15.4	
Other	2	7.4	0	0.0	2	15.4	
If so, for how many hours a week?							1.000
≤ 2	15	55.6	8	57.1	7	53.8	
3–6	5	18.5	2	14.3	3	23.1	
>6	7	25.9	4	28.6	3	23.1	
Discontinued physical activity on receiving the cancer diagnosis?							0.348
Yes	32	72.7	16	76.2	16	69.6	
Partially, but significantly	8	18.2	4	19.0	4	17.4	
No	1	2.3	1	4.8	0	0.0	
Other (already discontinued before receiving the cancer diagnosis)	3	6.8	0	0.0	3	13.0	
If so, reason for discontinuing?							—
Lack of time	11	27.5	6	30.0	5	25.0	
Fatigue	26	65.0	15	75.0	11	55.0	
Shame	0	0.0	0	0.0	0	0.0	
Not considered important	3	7.5	0	0.0	3	15.0	
Regarded as contraindicated	4	10.0	2	10.0	2	10.0	
Discouraged by coach	1	2.3	0	0.0	1	5.0	
Discouraged by general practitioner	9	22.5	6	30.0	3	15.0	
Discouraged by parents	3	7.5	1	5.0	2	10.0	
Others	9	22.5	6	30.0	3	15.0	
Who suggested the in-hospital exercise program?							—
Psychologist	6	14.3	5	23.8	1	4.8	
Oncologist	14	33.3	8	38.1	6	28.6	
Physiotherapist	29	69.1	19	90.5	10	47.6	
Other patients	6	14.3	2	9.5	4	19.1	
Leaflet, web	4	(9.1)	0	0.0	4	17.4	
I saw the gym myself	4	9.5	3	14.3	1	4.8	
Volunteers	3	7.1	1	4.8	2	9.5	

Overall, 27 patients (61%) reported having exercised regularly before their tumor was diagnosed, with no statistically significant difference between the two groups. Excluding three patients who reportedly did not exercise even before they were diagnosed with cancer, 32/41 patients (78%) reported discontinuing any form of physical activity when their disease was diagnosed, 8/41(20%) significantly reduced their level of exercise, and only one continued to exercise as before. The main reasons for discontinuing or limiting their exercise or sports were: fatigue (26/40); lack of time (11/40, partly due to frequent hospital visits); physical disabilities (amputation in one; problems leading to the inability to walk in five); and the presence of a central venous device (in two cases). Seventeen patients (43%) were discouraged from continued exercising by other people (general practitioners in nine cases, parents in three, other patients in four, and a sports coach in one) who regarded their tumor as a contraindication in itself.

Seventeen patients who had already attended the hospital gym before this study was launched (12 of whom reported not having exercised regularly or engaged in any sports before their tumor was diagnosed) were asked some additional questions referring to the previous period. Their motives for attending the gym were: to keep fit (8/17); to combat treatment-related side effects (3/17); to take their mind off their situation (12/17); to have an opportunity to socialize with their peers (2/17); to heal their relationship with their bodies (6/17). After starting to attend the hospital gym, they reported experiencing the following benefits: they felt physically fitter (8/17); they were better able to manage their anger (11/17), sadness (12/17), irritability (11/17), or anxiety (9/17); it helped to control side effects of treatment (nausea, vomiting), and it improved their relationships with peers (5/17). Fifteen of the 17 patients said they would come back to the hospital gym even after completing their cancer treatments, during their follow-up as outpatients (to get fitter before going to other gyms in five cases, to keep in touch with other patients in five, and because the in-hospital gym was seen as a more “protected” area in five).

### Results of the PedsQL-4.0 Generic Core and PedsQL Multidimensional Fatigue Scales

The median scores (and IQRs) for perceived emotional functioning were 85.0 (62.5–87.5) and 60.0 (45.0–75.0) in the GYM and No-GYM groups (*p* = 0.018), respectively. Specific items associated with this difference were: fear, sadness, sleeping difficulties, and uncertainty about the future, with median values of 100 (IQR: 75–100) vs. 75 (IQR: 50–100), 75 (IQR: 50–100) vs. 50 (IQR: 50–75), 100 (IQR: 50–100) vs. 75 (IQR: 50–100), and 100 (IQR: 50–100) vs. 50 (IQR: 25–75), respectively, for the GYM group vs. the No-GYM group.

For social functioning, the GYM group returned slightly higher scores than the No-GYM group for the items concerning getting along with peers (median 100 [IQR: 75–100] vs. 75 [IQR: 75–100]), and making friends (median 100 [IQR: 100–100] vs. 75 [IQR: 75–100]). The GYM group scored lower than the No-GYM group for the item about being able to do what peers can do (median 62.5 [IQR: 50–87.5] vs. 75 [IQR: 50–100]).

The GYM group also reported a slightly better psychosocial functioning than the No-GYM group (median 76.7 [IQR: 67.5–83.3] vs. 71.7 [IQR: 56.7–76.7]).

The median HRQoL scores obtained with the PedsQL Fatigue scale were lower for the GYM group than for the No-GYM group, except for the “general fatigue” dimension, which was a median 66.7 [IQR: 58.3–79.2] for the GYM group as opposed to 62.5 [IQR: 50–83.3] for the No-GYM group. On analyzing the different items, the median scores were higher in the GYM group than in the No-GYM group for perceived sleep quality (median 100 [IQR: 50–100] vs. 87.5 [IQR: 50–100]), and for fatigue in activities of daily living (median 100 [IQR: 75–100] vs. 87.5 [IQR: 75–10]). Overall, scores obtained in the questionnaires are reported in Table 2.

**Table 2:** Scores obtained in the questionnaires by single item and single dimension, and overall, for the whole cohort and by study group.

	**Overall**		**Gym**		**No-Gym**		
	***N*** **=** **44**		***N*** **=** **21**		***N*** **=** **23**		**Wilcoxon test's**
	**Median**	**IQR**	**Median**	**IQR**	**Median**	**IQR**	***p*-value**
**Physical health**							
1. It is hard for me to walk more than one block	100.0	75;100	100.0	75;100	100.0	75;100	
2. It is hard for me to run	50.0	25;100	50.0	0;75	50.0	25;100	
3. It is hard for me to do sports activity or exercise	50.0	25;75	50.0	0;75	50.0	25;75	
4. It is hard for me to lift something heavy	50.0	25;75	50.0	25;75	50.0	50:100	
5. It is hard for me to take a bath or shower by myself	100.0	75;100	100.0	100;100	100.0	75;100	
6. It is hard for me to do chores around the house	100.0	75;100	100.0	75;100	100.0	50;100	
7. I hurt or ache	75.0	50;100	62.5	50;100	75.0	50;100	
8. I have low energy	50.0	25;75	50.0	37,5;50	50.0	25;75	
Physical health dimension result^*^	65.6	56.3; 84.4	65.6	60.9; 71.8	65.6	50.0; 84.4	0.593
**Emotional functioning**							
1. I feel afraid or scared	75.0	50;100	100.0	75;100	75.0	50;100	
2. I feel sad or blue	50.0	50;100	75.0	50;100	50.0	50;75	
3. I feel angry	50.0	50;75	50.0	50;75	50.0	50;75	
4. I have trouble sleeping	75.0	50;100	100.0	50;100	75.0	50;100	
5. I worry about what will happen to me	75.0	50;100	100.0	50;100	50.0	25;75	
Emotional functioning dimension result^**^	70.0	50.0; 85.0	85	62.5; 87.5	60	45.0; 75.0	0.018
**Social functioning**							
1. I have trouble getting along with other adults	75.0	75;100	100.0	75;100	75.0	75;100	
2. Other adults do not want to be my friends	100.0	75;100	100.0	100;100	75.0	75;100	
3. Other adults tease me	100.0	75;100	100.0	100;100	100.0	75;100	
4. I cannot do things that others my age can do	75.0	50;100	62.5	50;88	75.0	50;100	
5. It is hard to keep up with my peers	75.0	50;100	75.0	50;100	75.0	50;100	
Social functioning dimension result^**^	85.0	70.0; 90.0	85	75.0; 90.0	85	70.0; 95.0	1.000
**School functioning**							
1. It is hard to pay attention at work or school	75.0	50;100	75.0	75;100	75.0	50;100	
2. I forget things	75.0	50;100	75.0	75;100	75.0	50;75	
3. I have trouble keeping up with my work or studies	75.0	50;100	75.0	50;100	75.0	25;75	
4. I miss work or school because of not feeling well	75.0	50;100	75.0	50;100	75.0	50;100	
5. I miss work or school to go to the doctor or hospital	50.0	25;50	25.0	25;50	50.0	25;75	
Scholar functioning dimension result^**^	70.0	55.0; 80.0	70	55.0; 80.0	60	40.0; 80.0	0.656
**General fatigue**							
1. I feel tired	50.0	25;50	50.0	25;50	50.0	25;75	
2. I feel physically weak	50.0	50;75	50.0	50;75	50.0	50;75	
3. I feel too tired to do the things I like to do	75.0	50;100	75.0	50;75	75.0	50;100	
4. I feel too tired to spend time with my friends	75.0	75;100	75.0	50;100	75.0	75;100	
5. I have trouble finishing things	75.0	50;100	75.0	50;100	75.0	50;100	
6. I have trouble starting things	75.0	50;100	50.0	50;100	75.0	50;100	
General fatigue dimension result^***^	66.7	54.1;83.3	66.7	58.3; 79.2	62.5	50.0; 83.3	0.866
**Sleep/rest fatigue**							
1. I sleep more often than usual	75.0	50;100	75.0	50;100	75.0	50;100	
2. It is hard for me to sleep all night	100.0	50;100	100.0	50;100	87.5	50;100	
3. I feel tired when I wake up in the morning	50.0	25;75	50.0	25;75	62.5	25;100	
4. I rest a lot	75.0	50;75	50.0	25;75	75.0	50;100	
5. I often sleep during the day	100.0	75;100	100.0	75;100	87.5	75;100	
6. I spend a lot of time in bed	75.0	50;100	50.0	25;75	75.0	50;100	
Sleep/rest fatigue dimension result^***^	70.8	54.1; 83.3	70.8	54.2; 79.2	75	50.0; 87.5	0.385
**Cognitive fatigue**							
1. It is hard for me to focus on something	50.0	25;100	50.0	0;100	50.0	25;50	
2. It is hard for me to remember what others tell me	25.0	0;100	0.0	0;100	50.0	0;100	
3. It is hard for me to remember what I have just heard	0.0	0;100	0.0	0;50	12.5	0;100	
4. It is hard for me to think quickly	50.0	0;100	25.0	0;100	50.0	0;100	
5. I have trouble remembering what I was thinking about	50.0	0;100	0.0	0;100	50.0	0;100	
6. I have trouble remembering more than one thing at a time	0.0	0;100	0.0	0;100	12.5	0;100	
Cognitive fatigue dimension result^***^	41.7	25.0; 58.3	37.5	16.7; 70.8	50	25.0; 58.3	0.357
**Global assessment**							
Physical health summary score^*^	65.6	56.3; 84.4	65.6	60.9; 71.9	65.6	50.0; 84.4	0.593
Psychosocial health summary score^**^	75	62.5; 80.0	76.7	67.5; 83.3	71.7	56.7; 76.7	0.132
Fatigue summary score^***^	59.7	50.0; 65.3	55.6	48.6; 63.9	61.8	50.0; 68.1	0.290

*IQR, Interquartile range*.

* *Physical health summary score is the same as the Physical health dimension, as this is a one-dimensional global assessment*.

** *Scales included in the global Psychosocial health summary score*.

*** *Scales included in the global Fatigue summary score*.

The correlations between total scores (for physical health, psychosocial health, and fatigue) were all from weakly to moderately positive, ranging between 0.51 (for physical health and fatigue) and 0.68 (for psychosocial health and fatigue) according to Pearson's correlation index (Supplementary Table 2; Supplementary Figure 1).

## Lessons Learned and Discussion

Physical activity has favorable effects on various levels of functioning in individuals during and after their treatment for cancer, encompassing aspects of QoL, mood symptoms, fitness level, muscle strength, body composition, and active attainment of social roles (5, 7, 8, 12, 25, 31, 32). A possible relationship between fitness level and the risk of mortality (4) and tumor recurrence (11, 33) has also emerged, and is worth exploring further.

The main findings of our study are: that a large proportion of children and adolescents with cancer abandon any physical activity without good reason when their disease is diagnosed; and that engaging in supervised exercise within the hospital improved patients' satisfaction with their emotional and social functioning.

When interviewed at the baseline, 97.5% of our patients who were active before being diagnosed with cancer reported a substantial decline in their exercising and sports activities afterwards. The main reasons mentioned for this included fatigue, lack of time, and physical disabilities. It is noteworthy that 42% of these patients had suspended their physical activities because they had been told, wrongly, that they were contraindicated for cancer patients. This is a common misconception, even among general practitioners, and worth reporting because its negative influence can be avoided with proper counseling on how safely such patients can exercise.

Our findings indicate that patients who attended the exercise sessions experienced a better emotional functioning than those who did not, obtaining significantly better scores especially for *fear, sadness, sleeping difficulties*, and *uncertainty about the future*. We know that the benefits of exercising and practicing sports go far beyond physical function endpoints alone, but there are still gaps in our understanding of how they benefit the emotional and social spheres, and resilience as well (13, 28). A key finding in a study on a large cohort of childhood cancer survivors was that vigorous exercise was associated with a lower risk of depression, somatization, and cognitive impairment (32). Training for a sports competition might symbolize patients' return to setting themselves healthy and challenging plans for their future. Endorphins, endocannabinoids, monoamines, and neurotrophins have all been implicated in the euphoric response to endurance running (34, 35), and may reinforce the biological rationale behind some of our findings. Other investigators have suggested that cardiorespiratory fitness may directly affect brain function, or that the psychological benefits of exercise result from improvements in sleep duration or quality (36).

Looking at the positive effects of exercising on *emotional* health, martial arts classes [which are part of our in-hospital sports programs (13)] have proved an interesting way to empower our patients emotionally too, improving their breathing and relaxation skills. A panel of experts strongly recommended the use of relaxation techniques or mindfulness to manage cancer-related fatigue (24).

A better *emotional* health and self-esteem can also relate to a better social functioning. Patients in our GYM group scored better on some *social* functioning items (e.g., *getting along with peers, making friends*). For adolescents especially, the benefits of engaging in sports might include regaining a sense of having a lively, properly-functioning body, enhancing relationship with peers and a spirit of independence. These findings are in line with research demonstrating positive relationships between physical activity and cognitive outcomes (25–27, 37). We know that adolescents with cancer are less active than their healthy peers (especially when in hospital), and that fatigue is more prevalent in this age group than in children, and might be particularly distressing (20, 38).

For the youngest children, including physical education in hospital routines may be important not only to promote physiological motor development (39, 40), but also to preserve or restore a sense of normality that cancer patients often lack. We know it is not easy to encourage small children to exercise, but we found that even 5-year-olds could take an active part in the workout sessions implemented in the present study.

On the other hand, our GYM group scored worse than the No-GYM group for the item *being able to do what peers can do*. In principle, everybody can find a suitable form of exercise to practice, though a delicate balance has to be found between patients' wishes and their capabilities (which may have been irreparably affected by their cancer). This may demand adapting certain exercises to patients with disabilities, or providing parallel psychological support in some circumstances. Asking patients to exercise may add to their frustrations if the demands placed on them exceed their expected capabilities, especially if levels of physical fitness are assumed to be similar to those a patient enjoyed before being diagnosed with cancer.

We have to aknowledge here that including patients both on and off therapy, which is a strength of our work, might also be a confounding factor (although patients on and off therapy were equally represented in the GYM and No-GYM groups) because patients no longer receiving treatment might have had more opportunities to interact and compare themselves with their peers, and this might have added to any frustration they experienced with their performance.

Our two groups differed in terms of cancer types: patients with brain tumors, who are known to suffer from neuromotor impairments (41), seemed more reluctant to attend the gym. The professionally-supervised programs adopted at our gym were essential to accommodate underlying organ system impairments. This meant that alternative, customized workouts could be used to exercise the less-impaired parts of a patient's body. That said, any lower limb impairment that prevented a patient from walking tended to be misconstrued as a reason for not exercising at all, even though such patients could work out effectively with their upper limbs and trunk.

Children attending the gym scored worse on fatigue, except for *general fatigue*. Analyzing the single items in the questionnaire revealed better median scores in the GYM group for perceived quality of *sleep* and *daily fatigue*. The impact of exercise on the severity of fatigue may differ, depending on the type of activity involved [aerobic, neuromotor, or combined exercises might be more effective than resistance exercises in improving a patient's fitness in terms of containing fatigue (24)], and on the intensity of the exercise. Given the diversity of cancer patient populations, recent publications have called for a more precise manipulation of training variables–such as volume, intensity, and frequency–to truly optimize clinically-relevant patient-reported outcomes (10). We did not homogeneously prescribe or record the training parameters adopted in our sample, nor even the overall number of training sessions attended by each patient over the 6-week period of observation. This is a limitation of our study, as it prevents us from knowing which type of exercise might be best for the study outcomes, or why some fatigue scores were worse for the GYM group.

In the recently-published paper on the randomized MUCKI trial (on 33 patients so far, which goes to show how challenging it is to design randomized studies), the authors found a positive effect of exercise on fatigue (42). We know that fatigue interferes with an individual's ability to engage in everyday life activities and social roles (including educational and work opportunities), and that it causes mood swings, impairs social relations, and lowers academic achievement (21, 22). One of the strengths of our study lies in that we examined several dimensions of HRQoL and fatigue, and the correlations between physical health, psychological functioning and fatigue as a whole. Our findings (Pearson's r for physical health and psychological functioning scores = 0.61) would suggest that individuals with a better neurocognitive functioning also experience a greater self-efficacy, which would make it easier for them to engage in physical activities. The direct correlation identified between a better perceived physical and emotional well-being and a lower perception of fatigue (Pearson's *r* = 0.68 and 0.51, respectively) probably stems from the fact that patients motivated to exercise and try to overcome their limits also had a more critical awareness of these limits and a better tolerance of their sense of fatigue.

Our study has limitations that need to be acknowledged. Patients were assigned to one of the two groups being compared not randomly, but based on their interest in joining the exercise program. The patients' reasons for attending the gym may have been positively or negatively influenced by their ability (or fear of inability) to cope with the workouts. They might also have been influenced by their attitude to sports before becoming ill (although a similar proportion of patients in the GYM and No-GYM groups had reportedly not exercised regularly before being diagnosed with cancer). Whatever their reasons, this may have affected their self-assessments and their opinion of the training program.

Our patients also formed a very heterogeneous group, with some on and some off therapy at the time of the study, and with ages ranging from 5 to 21 years. They had different types of solid tumor, and received different treatments, sometimes involving surgery and radiotherapy as well as chemotherapy (which was administered to all participants in the study). This means that any differences between the GYM and No-GYM groups may not be attributable to our exercise intervention alone, but also to other factors (e.g., changes in health status, different treatments).

The non-randomization of the intervention generated differences in the distributions between study groups of participants' personal and disease characteristics, particularly as regards their type of cancer. Due to the small number of participants involved, we could not usefully apply statistical tools like adjusted models or inverse probability of treatment weighting analysis to try to overcome this limitation.

A further limitation concerns the fact that no longitudinal data were available to enable any within-subject comparisons of the self-reported QoL outcomes for patients in the GYM group between before and after the 6-week exercise program.

## Conclusions

Our findings show the feasibility of promoting more systematic opportunities for young cancer patients to exercise, even while being actively treated, to improve their physical and psychosocial symptoms, their HRQoL, and especially their emotional functioning. Our results also suggest that many young patients with cancer, their parents, and their doctors still assume that a diagnosis of cancer makes it unsafe or unfeasible to exercise and practice sports, but this misconception can be overcome. Being aware that more systematic prospective trials are needed, we believe that our results could help shape the design of such future studies.

## Data Availability Statement

The raw data supporting the conclusions of this article will be made available by the authors, without undue reservation.

## Ethics Statement

The studies involving human participants were reviewed and approved by Fondazione IRCCS Istituto Nazionale Tumori, Milano (ref. INT 109/18). Written informed consent to participate in this study was provided by the participants' legal guardian/next of kin.

## Author Contributions

FS, FB, MMa, MC, MT, GG, and LV performed the literature review, contributed to the conception and design of the study, and contributed to data interpretation. FS, FB, MMu, MC, MT, AF, CM, MMa, and LV analyzed and interpreted the study data. FS and FB drafted the article. FS, FB, MMa, MC, and MMu substantially revised the article. FB, MMu, MC, GG, and LV acquired the data. FS, MMu, MC, MT, GG, CM, and LV recruited the study participants. All authors approved the final manuscript as submitted and agree to be accountable for all aspects of the work.

## Conflict of Interest

The authors declare that the research was conducted in the absence of any commercial or financial relationships that could be construed as a potential conflict of interest.

## Supplementary Material

Supplementary Figure 1 Scatter plots and spline curves of the correlation between the global assessment scores on the PedsQL-4.0 psychological (qolPSYCm) and physical (qolPHYSm) functioning scales, and the PedsQL-3.0-Fatigue (FATm) scale for the cohort as a whole.

Supplementary Table 1 Content of a typical 1-h supervised exercise session. The type and intensity of the exercises, and the duration of each phase were flexible, however, and tailored by the exercise professionals to patients' capabilities (and limitations), needs and preferences (yet not basing on cardiopulmonary exercise testing). Sessions were held in the gym adjacent to the inpatient care area.

Supplementary Table 2 Pearson's correlation indexes for PedsQL and PedsQL-Fatigue questionnaires. qolPSYCm, PedsQL-4.0 psychological scale; qolPHYSm, PedsQL-4.0 physical functioning scales; FATm, PedsQL-3.0-Fatigue scale.

## Abbreviations

HRQoL: health-related quality of life
IQR: Interquartile range
QoL: quality of life.
