# Supplementary figures and images for: Positive Impact of Organized Physical Exercise on Quality of Life and Fatigue in Children and Adolescents With Cancer
Source: Front Pediatr. 2021 Jun 7;9:627876. doi: 10.3389/fped.2021.627876 (PMC8215206; doi:10.3389/fped.2021.627876)

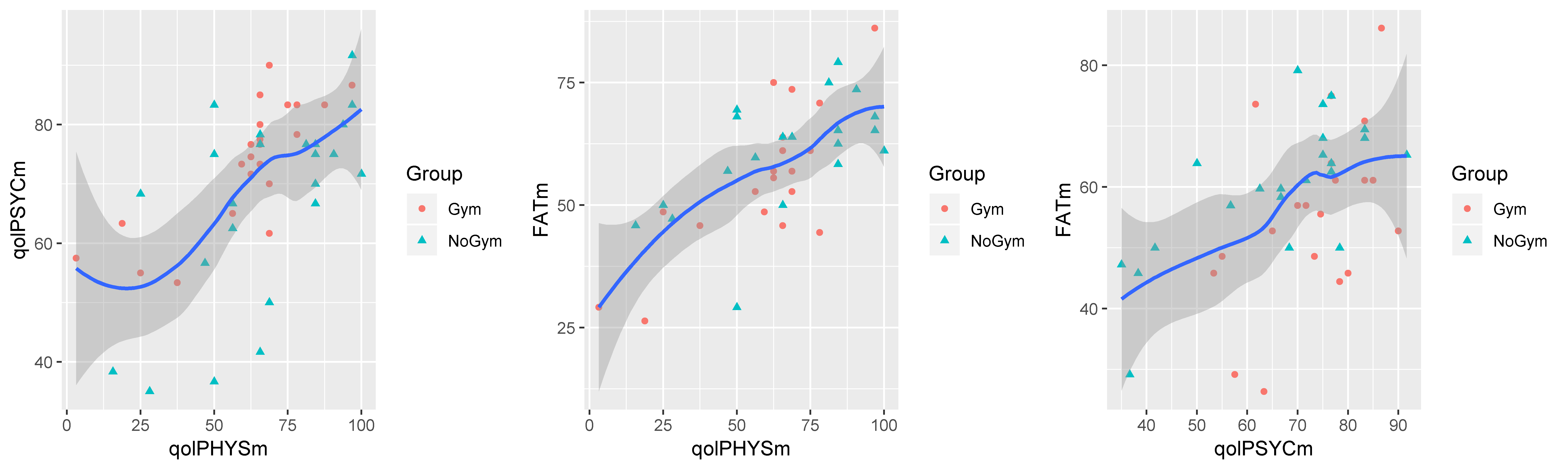

Supplement: Supplementary Figure 1 — Scatter plots and spline curves of the correlation between the global assessment scores on the PedsQL-4.0 psychological (qolPSYCm) and physical (qolPHYSm) functioning scales, and the PedsQL-3.0-Fatigue (FATm) scale for the cohort as a whole. [file Image_1.PNG]
