# Supplementary material for: Positive Impact of Organized Physical Exercise on Quality of Life and Fatigue in Children and Adolescents With Cancer
Source: Front Pediatr. 2021 Jun 7;9:627876. doi: 10.3389/fped.2021.627876 (PMC8215206; doi:10.3389/fped.2021.627876)
Supplement: Supplementary Table 1 — Content of a typical 1-h supervised exercise session. The type and intensity of the exercises, and the duration of each phase were flexible, however, and tailored by the exercise professionals to patients' capabilities (and limitations), needs and preferences (yet not basing on cardiopulmonary exercise testing). Sessions were held in the gym adjacent to the inpatient care area. [file Table_1.DOCX]

**Supplementary table 1**. Content of a typical 1-hour supervised exercise session. The type and intensity of the exercises, and the duration of each phase were flexible, however, and tailored by the exercise professionals to patients’ capabilities (and limitations), needs and preferences (yet not basing on cardiopulmonary testing). Sessions were held in the gym adjacent to the inpatient care area.

| **Exercise modality** | **Duration per session** | **Notes** |
| --- | --- | --- |
| Free body warm-up exercises | 5-10’ | thoracic and scapulohumeral joint mobility exercises |
| Aerobic exercises | 20-30’ | variable intensity, with exercise bike or treadmill |
| Bodyweight workout | 20-25’ | including exercises for central stability, scapulohumeral joint stability, dynamic stability, strength and balance, performed mainly with resistance bands |
| Relaxation exercises and muscle stretching | 5-10’ |  |
