# Supplementary material for: Positive Impact of Organized Physical Exercise on Quality of Life and Fatigue in Children and Adolescents With Cancer
Source: Front Pediatr. 2021 Jun 7;9:627876. doi: 10.3389/fped.2021.627876 (PMC8215206; doi:10.3389/fped.2021.627876)
Supplement: Supplementary Table 2 — Pearson's correlation indexes for PedsQL and PedsQL-Fatigue questionnaires. qolPSYCm, PedsQL-4.0 psychological scale; qolPHYSm, PedsQL-4.0 physical functioning scales; FATm, PedsQL-3.0-Fatigue scale. [file Table_2.DOCX]

|  | **Pearson Correlation Coefficients** | | |
| --- | --- | --- | --- |
|  | **qolPHYSm** | **qolPSYCm** | **FATm** |
| **qolPHYSm** | 1.000 | 0.613 | 0.681 |
| **qolPSYCm** | 0.613 | 1.000 | 0.515 |
| **FATm** | 0.681 | 0.515 | 1.000 |

**Supplementary Table 2.** Pearson’s correlation indexes for PedsQL and PedsQL-Fatigue questionnaires. Abbreviation: qolPSYCm, PedsQL-4.0 psychological scale; qolPHYSm, PedsQL-4.0 physical functioning scales; FATm, PedsQL-3.0-Fatigue scale.
